# Quality of Care and Job Satisfaction in the European Home Care Setting: Research Protocol

**DOI:** 10.5334/ijic.2519

**Published:** 2016-08-31

**Authors:** Liza Van Eenoo, Henriëtte van der Roest, Hein van Hout, Anja Declercq

**Affiliations:** 1 LUCAS, KU Leuven, Leuven, Belgium; 2 Department of General Practice and Elderly Care Medicine, VU University Medical Center, Amsterdam, The Netherlands

**Keywords:** Europe, older people, community care, care dependent elderly, quality of care, job satisfaction, integrated care, research protocol

## Abstract

**Introduction:**

Since the European population is ageing, a growing number of elderly will need home care. Consequently, high quality home care for the elderly remains an important challenge. Job satisfaction among care professionals is regarded as an important aspect of the quality of home care.

**Aim:**

This paper describes a research protocol to identify elements that have an impact on job satisfaction among care professionals and on quality of care for older people in the home care setting of six European countries.

**Methods:**

Data on elements at the macro-level (policy), meso-level (care organisations) and micro-level (clients) are of importance in determining job satisfaction and quality of care. Macro-level indicators will be identified in a previously published literature review. At meso- and micro-level, data will be collected by means of two questionnaires utilsed with both care organisations and care professionals, and by means of interRAI Home Care assessments of clients. The client assessments will be used to calculate quality of care indicators. Subsequently, data will be analysed by means of linear and stepwise multiple regression analyses, correlations and multilevel techniques.

**Conclusions and Discussion:**

These results can guide health care policy makers in their decision making process in order to increase the quality of home care in their organisation, in their country or in Europe.

## Introduction

The European population is ageing. The proportion of persons in Europe of 65 years and older will increase from 16% in 2010 to 27.8% in 2050. Consequently, by 2050, one out of five people in Iceland, and one out of three people in Germany and Italy will be 65 years of age or older [1]. Subsequently, the proportion of people aged of 80 years or older is expected to increase from 4.1% in 2010 to 10% by 2050 [1,2].

Associated with this, the total number of care dependent older people will increase [3]. The Survey of Health, Ageing and Retirement in Europe (SHARE) concluded in 2004 that approximately 20% of the European population aged 65 years and older were physical dependent, which was defined as ‘cannot perform activities of daily living due to physical limitation’ [3]. Of those dependent older persons, 42% received formal care. Two out of three of the formal care users (67%) received formal care at home, with a variation between 44% in Belgium and 83% in Italy [3].

This growing number of care dependent older persons, along with the fact that the European care policy makers are looking for sustainable ways to organise health care [4], and with the wish of older persons to remain in their home environment as independently as possible and for as long as possible [5], will cause a growing number of care dependent older persons in the European home care setting.

Consequently, providing high quality care for care dependent older people in the European home care setting remains a significant challenge. There is a large variation in funding, organisation, and delivery of community based care in Europe [6,7,8]. The aim of this study is to identify which elements have an impact on the quality of home care.

## Conceptual framework

**Figure 1:**
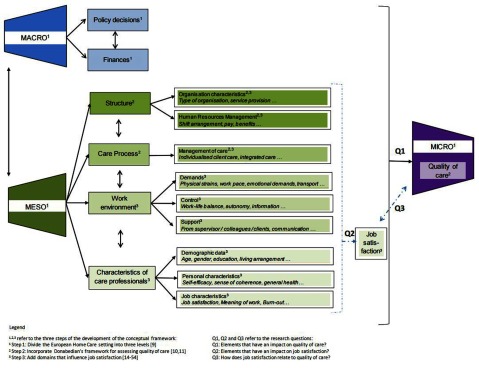
Conceptual framework of the study protocol ‘Quality of care and job satisfaction in the European home care setting’.

In order to research this aim, we first developed a conceptual framework (Fig. 1) in three steps.

### Step 1: Divide the European Home Care setting into three levels

The World Health Organisation defined the health care system as ‘the system which encompasses all the activities whose primary purpose is to promote, restore, or maintain health’ [9]. These systems are remarkably expansive and include clients and their families, health care workers and caregivers within organisations and in the community, and the health policy environment in which all health related activities occur [9]. One strategy to organise thinking about health care systems, and thus also about the European home care system, is to divide these complicated networks into strata or levels: the micro, the meso and the macro level [9]. The micro level refers to the client interaction level. At meso level, the health care organisation coordinates the delivery and evaluates the quality of the services provided. The organisation has a responsibility to unite care professionals, provide them with the expertise and tools they need to perform their roles in managing clients, and link to community resources [9]. At the macro or policy level, overall values, principles and strategies for health care are developed, and decisions concerning resource allocation are made [9]. Each of these three levels interacts and dynamically influences the other two. When micro-, meso- and macro-levels work effectively within themselves, and successfully function in relation to each other, health care is efficient and effective and clients experience better health. Dysfunction within and among the levels creates waste and ineffectiveness [9].

Therefore, to examine quality of care of the complicated network of the European home care system, we divided the system into three levels in the first step of the development of our conceptual framework (see step 1 in Fig. 1). The micro level refers to the home care clients, the meso level to the home care organisations and their care professionals and the macro level to the home care policy.

### Step 2: Incorporate Donabedian’s framework for assessing quality of care

**Figure 2:**
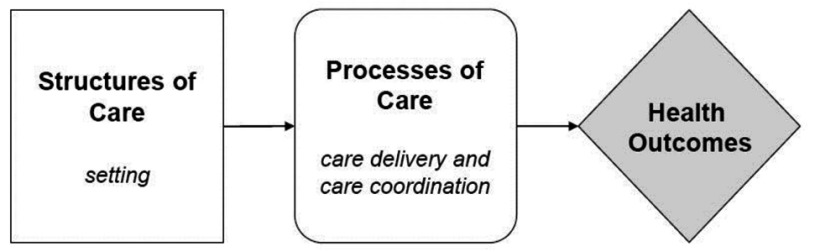
Donabedian’s Quality Framework [11].

Since the aim of the study is to identify elements that have an impact on the quality of home care, we based the second step of the development of our conceptual framework on Donabedian’s framework for assessing the quality of care (see Fig. 2) [10,11]. This framework shows that for assessing the quality of care, the structural characteristics and the care processes are two important pillars. Structural characteristics can be defined as the physical and organisational aspects of the settings in which care occurs. This includes aspects of material resources (such as facilities, equipment and money), of human resources (such as the number and qualification of personnel) and of organisational structure (such as medical staff organisation, methods of peer review and methods of reimbursement). The care processes denote what is actually done in giving and receiving care [10,11]. We have incorporated Donabedian’s framework into our conceptual framework, by adding the ‘structural characteristics’ and ‘care processes’ at the meso level (see step 2 in Fig. 1). At the micro level or client level, health outcomes will be measured as an indicator of quality of care.

### Step 3: Add domains that influence job satisfaction

Since care professionals are part of the home care organisation [9] and essential for delivering (high quality) client care [12], we searched for further literature on the characteristics of care professionals in a third step. An important intrinsic characteristic of care professionals that is associated with quality of care, is job satisfaction [13,14,15,16,17]. In hospitals [13] as well as in nursing homes [14] a significant association between job satisfaction and quality of care as assessed by care professional has been shown (r=0.47 and r=059 respectively). Research also shows that job satisfaction is key to worker retention [15,16,17]. High turnover rates are a serious concern in long term care [12,14] and are linked with poor quality of care or with no care at all [16,18]. Based on these findings, we hypothesise that job satisfaction in care professionals must be sufficiently high in order to provide good quality of care. A literature review on job satisfaction among care professionals working in elderly home care setting, resulted in 14 domains that have an impact on job satisfaction [14,15,16,17,18,19,20,21,21,23,24,25,26,27,28,29,30,31,32,33,34,35,36,37,38,39,40,41,42,43,44,45,46,47,48,49,50,51,52,53,54]. These domains can be categorised into personal characteristics, job-related characteristics, organisational characteristics and client outcomes.

- Personal characteristics
  - Demographic data such as age, gender, education and financial situation of the care professional.
  - Parameters of the personal situation such as social support outside the workplace, mental and physical health status.
- Job characteristics
  - General job characteristic such as seniority, absenteeism and self-efficacy.
  - Job security.
  - Meaning of work.
  - Commitment to the workplace and commitment to the profession.
  - Job outcomes such as intention to leave, burn-out and work-related stress.
- Organisational characteristics
  - Structural characteristics of the organisation, such as location and type.
  - Work organisation structure, such as work shift arrangement, opportunities for development and satisfaction about scheduling and payment.
  - Elements of the management of care or the care processes, such as the extent of individualised patient care and the extent of integrated care.
  - Work environment characteristics
    - Demands at the workplace, such as physical strain, and demands related to role conflict and ambiguity, and quantitative, emotional and cognitive demands.
    - Control at the workplace such as work-life balance, influence at work and degree of freedom at work.
    - Support and relationships at work, such as support from supervisors, colleagues and patients, social inclusiveness and offensive behavior and meetings.
- Client outcomes
  - In order to incorporate these domains into our conceptual framework, we added ‘work environment’ and the ‘characteristics of the care professionals’ to the meso level (see step 3 in Fig. 1). The other domains were already mentioned by the second step. Since we will focus on job satisfaction, we added an additional box ‘job satisfaction’ to indicate that this is a central variable in our study.

In summary, in order to understand why some organisations within the context of health care systems provide better quality of care than others, we hypothesise that the community care policy (macro level), the structure and the care processes of community care organisations (meso level) and client outcome (micro level) are equally important. Studies that directly link these three pillars of community care to quality of provided care have not yet been conducted to our knowledge. Additionally, we hypothesise that job satisfaction must be sufficiently high in order to provide good quality of care. Little is known about the linkages between job satisfaction and quality of care in the home care setting. Additionally, studies that examined the relationship between quality of care and job satisfaction, mostly examined the quality of care from the point of view of the care professional. In this study, we will measure quality of home care by means of the health outcomes of the clients. Al this taken into consideration, we formulated the following research questions:

1. Which elements of community care for care dependent older persons across Europe, regarding community care policy (macro level), community care organisations and community care professionals (meso level) have an impact on the quality of care (micro level)? (see Q1 in Fig. 1)
2. Which elements of community care for care dependent older persons across Europe, regarding community care organisations and community care professionals (meso level) have an impact on the job satisfaction among the care professionals? (see Q2 in Fig. 1)
3. How does job satisfaction relate to the quality of care? (see Q3 in Fig. 1)

## Methods

### Study design

In order to examine the research questions, we will use an observational, prospective, quantitative design. Longitudinal data (3 waves) on the health status will be collected from individual clients to establish the delivered quality of care (micro level). Cross-sectional data will be collected on characteristics of care professionals and of community care organisations (meso level). Additionally, characteristics of care systems at national and local levels (macro level) will be described and compared.

### Ethics statement

This study was conducted within the IBenC project (Identifying best practices for care-dependent elderly by Benchmarking Costs and outcomes of community care [55] (FP7, grant no. 305912).

Medical ethical approvals for the study have been provided by all the competent legal Ethic Boards in each data collecting country.

### Setting and participants

#### Macro level – community care policy

This study is focused on the community care policy of six European countries: Belgium (Flanders), Finland, Germany, Iceland, Italy and the Netherlands. Home care or community care is defined as ‘care provided at home by professionals’ [55].

#### Meso level – community care organisations and care professionals

Each participating country includes at least three community care organisations. A community care organisation is defined as a professional care organisation that offers nursing care (activities of nurses that are of a technical, supportive or rehabilitative nature), personal care (assistance with activities of daily living (ADL) such as dressing, feeding, washing and toileting and getting in or out of bed) and/or domestic care (help with instrumental activities of daily living (IADL), such as shopping, food preparation, housekeeping, transportation, taking medication and financial administration) in the community [55].

The home care professionals are defined as all care professionals of the included home care organisations, namely nurses and second level nurses, social workers, home health aides, managers with a leading position, supporting administrative staff and other care professionals who provide care to the clients at home.

#### Micro level – clients

Based on a power analysis of 80%, bearing in mind that a drop-out of 15% can be expected, we can conclude that each organisation should include at least 153 clients [55,56,57]. Consequently, each country should include at least 459 clients and in total we should include at least 2750 clients.

The clients included for this study are care dependent older persons who receive long-term home care from the included home care organisations and professionals. The clients need to be (1) 65 years or older and (2) care dependent, which is defined as receiving home care from a least one professional home care organisation. To include clients who need long-term home care we will ask the care professionals that they include clients of whom they expect that the client will receive home care for a period of least 6 months. Clients having a terminal illness will be excluded in this study.

### Instruments

#### Macro level – community care policy

In order to characterise the macro level, we already performed an evidence-based literature study [58]. Hereby, we distinguished the following dimensions to describe community care delivery on the macro level:

- Population of the country
- Governmental expenditures
- Sources of community health services funding
- Governmental vision on community care
- Governmental regulation on the organisation of community care
- Provision of community care: organisations and professionals
- Eligibility criteria for and equity in receiving care
- Involvement of informal care

Relevant indicators within these dimensions were identified, collected, complemented and summarised.

#### Meso level – community care organisations and care professionals

In order to collect reliable data across the participating countries on the meso level, we developed two questionnaires on evidence-based literature. A first questionnaire to collect data on the structure and the care processes of the meso level (questionnaire on the characteristics of the home care organisations) and a second questionnaire to collect data on the work environment and the characteristics of the care professionals (questionnaire on the characteristics of the care professionals). In order to ensure the face validity of both questionnaires, an English draft questionnaire was reviewed by three managers of home care organisations in Belgium and the Netherlands and by seven academic experts from the six participating countries. Questions were tested for applicability to the community care setting and the different countries, clarity, and completeness. The questionnaires were adjusted based on the comments and subsequently checked for language by a native English speaker. In order to ensure standardisation of the questionnaires across the six countries, the final questionnaires were translated and back-translated in the different languages.

##### Questionnaire on the characteristics of the home care organisations

The questionnaire on the characteristics of the home care organisations was developed on evidence-based literature on community care organisations and quality of care [59,60,61,62,63,64,65]. Elements concerning integrated care were included based on the theoretical framework of the Care Coordination Atlas [65]. The final questionnaire on the characteristics of the home care organisations is structured along four main themes:

1. The structure of the organisations: e.g. type of the home care organisation, ownership of the organisation, location and size of the service region.
2. The caseload of the organisations: e.g. total number of clients, number of clients who are 65 years or older and total number of client visits during the previous calendar year.
3. The characteristics of care professionals: e.g. disciplines of care professionals, number and FTE per discipline, educational level of the care professionals, turn-over rate and education and training of the care professionals.
4. The organisational processes: e.g. professionals on call, care management, referral and transfer procedures, meetings and accountability.

##### Questionnaire on the characteristics of the home care professionals

The questionnaire on the characteristics of the home care professionals was developed on evidence-based literature on job satisfaction among care professionals in the elderly home care setting [14,15,16,17,18,19,20,21,21,23,24,25,26,27,28,29,30,31,32,33,34,35,36,37,38,39,40,41,42,43,44,45,46,47,48,49,50,51,52,53,54]. This questionnaire mainly consists of pre-existing validated questions and scales: the Copenhagen Psychosocial Questionnaire [66,67], the Copenhagen Burnout Inventory [68], the impact subscale of the Job Role Quality survey [69], the Intention-to-turnover scale [70], the Scheduling Dissatisfaction Scale [51], the Physical Workload Scale [71] and the Individualised Care Scale-nurse [72,52]. Additionally, items on demographic characteristics, and questions concerning the social situation and job characteristics were added.

###### Copenhagen Psychosocial Questionnaire (COPSOQ)

The Copenhagen Psychosocial Questionnaire (COPSOQ I) has been developed in 1997 by the Psychosocial Department, National Institute of Occupational Health at Copenhagen Denmark (NRCWE, http://www.arbejdsmiljoforskning.dk/en) as a tool to assess a broad range of psychosocial work environment factors [66]. In 2007, the questionnaire was updated resulting in COPSOQ II. The COPSOQ II contains 22 relevant dimensions and questions had a four or five response options. The COPSOQ concept is a valid and reliable tool (Cronbach’s α between 0.73 and 0.89) [66,67] for workplace surveys, analytic research, interventions, and international comparisons.

Since the scale values of the short version of the COPSOQ II correspond very well with the scale values of the long version (r between 0.73 and 1.00) [73] we decided in consultation with the COPSOQ network, to use the short-length version of the COPSOQ II for our study. We added the COPSOQ I subscales degrees of freedom, job insecurity and salary, also important aspects of job satisfaction according to the literature review. Since it is also recommended to measure the ‘organisational social capital’ (features of social relationships that facilitate collective action for mutual benefits) [74], we include the COPSOQ I subscale ‘social community at work’ [74].

The level of job satisfaction among the care professionals will be measured by the ‘job satisfaction’ subscale consisting of one item: “Regarding your work in general, how pleased are you with your job as a whole, everything taken into consideration?” (Very satisfied – Satisfied – Unsatisfied – Very unsatisfied).

###### Copenhagen Burnout Inventory

The Copenhagen Burnout Inventory (CBI) is a valid and reliable instrument (Cronbach’s α = 0.87, 0.87 and 0.85) developed as part of the PUMA study in order to examine the burnout of employees working in the field of human service [20,68]. The total CBI has been included in the questionnaire.

###### Impact subscale of the Job Role Quality Survey

We will use the Impact-subscale of the Job Role Quality survey, developed by Marshall et al [69] to measure to which extent the job is rewarding for the care professionals.

###### Intention-to-turnover scale

The intention-to-turnover will be assessed by two items of the Michigan Organisational Assessment Questionnaire: (1) I will probably look for a new job in the next year and (2) I often think about quitting [70].

###### Scheduling Dissatisfaction Scale

The Scheduling Dissatisfaction Scale (SDS) is a 7-item Likert scale to measure the level of (dis)satisfaction of the work schedule of nurses. The SDS is a reliable instrument (Cronbach α=0.88) according to active nurses from acute care, long-term care, community health, primary care and home care [51].

After consultation with Prof. Stewart, we will use a modified 6-item SDS in our study (Cronbach’s α = 0.86) (N.J. Stewart, personal communication), bearing in mind that the SDS has not been used for other care professionals than nurses up to date.

###### Physical workload scale

The 4 item physical workload scale (PWS) was developed to assess the physical workload [71]. Cronbach’s α of the scale is 0.84 [71].

###### Individualised Care Scale

The Individualised Care Scale – nurse version (ICS-nurse) has been developed in order to assess the level of provision of individualised patient care from the nurses’ point of view [52,72]. Individualised care is defined as a type of nursing care delivery which takes into account patients’ personal characteristics in their clinical condition, their personal life situation and their preferences promoting patient participation in decision making [75]. The ICS-nurse is a two-part instrument measuring the support of patient individuality in specific nursing activities (ICS-A-nurse) and the perceptions of individuality in care provision (ICS-B-nurse).

After permission of and in consultation with Dr. Suhonen, we will use the ICS-B-nurse scale in our study to examine to what extend the care professionals provide individualised care in the care professionals point of view. The Cronbach’s α for the ICS-B-nurse is 0.90 [72].

#### Micro level – clients

The health status of the individual clients will be measured by means of the interRAI Home Care (HC) instrument. The interRAI HC instrument is a valid and reliable comprehensive geriatric assessment that can be used to collect data on frail elder persons in the community care setting in a structured and standardised way [57,76,77,78]. Based on the interRAI HC assessments, twenty three interRAI home care quality indicators can be calculated, with risk-adjustment. These risk-adjusted quality indicators are multi-dimensional, focusing on function, clinical complexity, social life, distress and service use. They provide new opportunities to identify international best practices. [79,80]. Additionally, two summary quality scales, encompassing twenty of the twenty-three quality indicators, were identified in order to evaluate the quality of provided care: the interRAI Home Care Independence Quality Scale and the interRAI Home Care Clinical Balance Quality Scale [79,80].

### Procedures

#### Macro level – community care policy

Country specific descriptions and macro indicators on community care were reviewed by an academic expert in each of the countries. Principal investigators from the participating IBenC countries checked if the information was correct, sufficiently detailed, up to date and replaced incorrect information if needed.

#### Meso level – community care organisations and care professionals

Per included organisation, one questionnaire on the characteristics of the home care organisations will be filled out by a manager with a leading position. This questionnaire mainly consists of multiple choice questions. It can be filled out online or on paper and it will takes between 45 and 60 minutes to fill out the questionnaire.

All care professional working within the participating community care organisation will be asked to fill out the questionnaire on the characteristics of the care professionals. It can be filled out online or on paper and it takes about 30 minutes. All care professionals are asked to sign an informed consent agreement. Refusing to participate will not have an impact on the working situation of the care professionals. All data on care professionals will be collected and analysed in an anonymous way.

#### Micro level – clients

The data collection on the clients’ health status by means of the interRAI HC instruments will be performed by trained community care professionals or trained research nurses. The training will be given at the beginning of the study and consists of learning how to fill out the interRAI HC instrument and of information on the study protocol. If needed, new care professionals will be trained during the study.

Depending of the regulations of the participating countries, an informed consent will be signed by the clients or their close relative before starting the data collection. Refusing to participate will not affect the care offered to the clients.

In order to calculate quality of home care indicators, longitudinal data is needed [79,80]. Therefore, clients will be visited at home at baseline, after six months and after twelve months as part of routine care. Additional information on the client’s status will be derived from the client’s patient files. The interRAI HC assessments have to be performed by, or in conjunction with, the home care professional who is responsible for the care of the client and who is able to assess the functioning of the care recipient.

All data on the clients will be collected and analysed in an anonymous way.

### Analyses

The macro level has already been analysed by means of a literature review [58]. This resulted in country specific dimensions on community care that allow for comparisons between care policies within and between the six countries.

For the analyses at the meso and micro level, quantitative data will be collected. The data on the meso level will be derived from the questionnaires on the characteristics of the home care organisations and of the home care professionals. The data on the micro level will be derived from the interRAI HC assessments. Consequently, we will have three quantitative databases. Since the included clients will receive care from the included care professionals who belong to the included home care organisations, multilevel analyses (or hierarchical modeling) are appropriate. This analytical approach allows for the simultaneous examination of the effects of group-level and individual-level variables on individual-level outcomes [81]. Multilevel analysis involves two-level data structures, but extensions to multiple levels are possible [82].

Preliminary, descriptive analyses on data on the home care organisations, the home care professionals and the clients included in our study will be performed, in order to determine whether there are significant differences between the samples from the different countries and organisations. For example, care dependence may vary. When measuring quality of care, perceived differences will be taken into account by means of risk adjustment [79,80].

In order to answer the first research question ‘Which elements of community care for care dependent older persons across Europe have an impact on the quality of care (Q1)’, risk-adjusted interRAI home care quality indicators will be calculated in order to identify best practices [79,80]. By means of percentiles, the best practices (P95) and the worst practices (P5) can be determined and grouped. By aggregating the quality of care indicators and the data of the care professionals to the organisation level, it will be possible to compare elements of the best practices with the worst practices on the meso level. During these analyses, it is possible that differences emerge which cannot be explained by the variables at the meso or micro level. The variables at the macro level may help to explain these differences.

The second research question ‘Which elements of community care for care dependent older persons across Europe have an impact on job satisfaction among care professionals (Q2)’ will be analysed by means of linear regression analyses, stepwise multiple regression analyses and multilevel techniques.

The third goal of our study is to determine whether there is a relationship between job satisfaction and quality of care (Q3). In this part of the analysis, we will make use of Spearman’s rank correlation and regression analyses in order to evaluate whether job satisfaction has a direct correlation or an intermediated association with quality of care.

All data will be analysed using SPSS© version 22.

## Discussion

This article describes the research protocol of a study with the overall aim to identify community care elements that have impact on quality of care for care dependent older people and have impact on job satisfaction among care professionals in the European home care setting. An observational, prospective, quantitative design is being used.

Both quality of care as job satisfaction are central variables in this study. Both variables are first individually analysed as outcome measures. Subsequently, we will analyse how job satisfaction is associated to quality of care. We expect that the job satisfaction among care professionals must be sufficiently high in order to provide good quality of care. Job satisfaction could be a direct factor on quality of care, but it also might very well be a mediating factor on client outcomes. Both hypotheses will be explored.

Since there are other national and international studies that used the same questionnaires such as the COPSOQ [66,76] or the interRAI HC instrument [56,57] , comparisons with other (health care) settings and care dependent elder people from previous studies are possible. However, this also raises the issue of the lack of validated questionnaires to assess organisational characteristics. By carefully developing the questionnaire on the characteristics of the organisations within this study, we hope to collect reliable data in a standardised way. Only in Belgium and the Netherlands, home care managers were involved in the development of the questionnaire. However, seven academic expert from the six participating countries did review the questionnaire.

Another potential limitation of the study concerns the participating home care professionals. Not all types of community care professionals will be represented. We will include larger home care organisations. As a consequence, independent nurses will not participate.

Another potential limitation of the study is the generalisability of the future results. The study is about community care in six European countries, all of which are part of Western Europe. The results may not be applicable to other parts of Europe. However, the methods used could be applied globally. Furthermore, only a part of each country and only a limited number of organisations per country will be participating in the research. Care organisations were not recruited based on representativeness, but on variety in type.

## Conclusion

Providing high quality care for care dependent older people in the European home care setting remains a significant challenge. To measure quality of care, we hypothesise that we need information on macro (policy), meso (home care organisations) and micro (clients) level. Additionally, we hypothesise that the job satisfaction among care professionals must be sufficiently high in order to provide good quality of care. Consequently, during the study described in this protocol, we will examine elements that contribute to higher job satisfaction and to higher quality of care. Also the relationship between job satisfaction and quality of care will be analysed.

Since it is important to invest in quality of care for care dependent older persons, the results of this study will give added values to policymakers on different levels. By examining the local home care organisations based on the study results, regional policymakers can determine policy priorities for increasing the quality of care in their own organisations. National and international policy makers can use the study results in order to develop better home care regulations in their own country and across the European home care settings. Finally, evidence-based guidelines and interventions can be developed. Further research should evaluate these evidence-based guidelines and interventions.
